# The cytotoxicity of gomesin peptides is mediated by the glycosphingolipid pathway and lipid-cholesterol interactions

**DOI:** 10.1038/s41420-025-02817-x

**Published:** 2025-11-21

**Authors:** Isabel Fernandez-Carrasco, Javier Moral-Sanz, Sergey Kurdyukov, Èlia Obis, Lissy Maike Hartmann, Silvia Carina Magalhães Novais, Matthew A. Waller, Naomi McKinnon, Felicity Chung, Francisco Javier Salazar Castejón, Daniel P. Rainho, Zoltan Dekan, Thomas Kremsmayr, Bernhard Jandl, Kristina Eleršič Filipič, Reinald Pamplona, Mariona Jové, Manuel A. Fernandez-Rojo, Gregor Anderluh, Markus Muttenthaler, Paul F. A. Alewood, Jan Procházka, G. Gregory Neely, Evelyne Deplazes, Maria P. Ikonomopoulou

**Affiliations:** 1 Madrid Institute for Advanced Studies in Nutrition (IMDEA Nutrition), Madrid, Spain; 2 Dr. John and Anne Chong Lab for Functional Genomics, Charles Perkins Centre and School of Life & Environmental Sciences, The University of Sydney, Sydney, NSW Australia; 3 Department of Experimental Medicine, Lleida Biomedical Research Institute (IRB Lleida), University of Lleida (UdL), Lleida, Spain; 4 School of Chemistry and Molecular Biosciences, The University of Queensland, St. Lucia, QLD Australia; 5 Czech Centre for Phenogenomics, Institute of Molecular Genetics of the Czech Academy of Sciences, Vestec, Czechia; 6 Institute for Molecular Bioscience, The University of Queensland, Brisbane, QLD Australia; 7 Institute of Biological Chemistry, University of Vienna, Vienna, Austria; 8 Department of Molecular Biology and Nanobiotechnology, National Institute of Chemistry, Ljubljana, Slovenia; 9 Frazer Institute, Translational Research Institute, The University of Queensland, Woolloongabba, QLD Australia; 10 School of Life Sciences, University of Technology Sydney, Ultimo, NSW Australia

**Keywords:** Melanoma, Biologics

## Abstract

Gomesins (AgGom and HiGom) are therapeutically promising spider-derived peptides that target a specific phospholipid composition (3PC:1PS:1Chol) to disrupt melanoma cell membranes and induce cytotoxicity. Their antiproliferative properties are interrelated to lipid metabolism, particularly glycosphingolipid biosynthesis. We used lipidomics, CRISPR/Cas9 knockout screening, molecular and biophysical experiments, followed by xenograft melanoma animal studies to demonstrate that gomesins target the glycosphingolipid pathway via inhibition of the ST3GAL5 gene. Notably, the addition of cholesterol reduced the cytotoxicity of gomesins, which may explain why melanoma cells with lower cholesterol levels than neonatal foreskin fibroblasts are more sensitive to gomesins. We propose that gomesins bind to melanoma CRAC domains, restricting intracellular cholesterol and thereby enhancing their cytotoxicity. In line with this hypothesis, cholesterol sequestration and the disruption of lipid raft microdomains enhanced the cytotoxic effects of gomesins in melanoma cells, whereas adding cholesterol in membrane permeability and proliferation assays reduced the effects of gomesin treatment. Taken together, this study highlights the specific role of cholesterol and the glycosphingolipid pathway in melanoma cells, paving the way for new strategies in targeted melanoma therapies.

## Introduction

Skin cancers are the most frequently diagnosed cancers globally, with around 1.5 million cases reported in 2020 and a projected incidence increase of over 50% by 2040 [1]. Melanoma, a particularly aggressive form of skin cancer, has a complex etiology driven by both genetic and environmental factors [2]. A *BRAF* mutation is present in 40–50% of all melanoma cases, with the most common one, *BRAFV600E*, involving a glutamic acid-to-valine substitution at codon 600 [3]. The high metastatic melanoma potential, significant morbidity, and ability to develop chemotherapy resistance contribute to ineffective therapeutic outcomes [1]. BRAF/MEK-targeted therapies and immune checkpoint inhibitors have remarkable success against melanoma [2]. However, these treatments are often associated with toxicity, and nearly all patients eventually develop resistance, leading to tumor relapse [2]. Thus, there remains a need for innovative therapeutic strategies and new drugs to improve survival rates in metastatic or advanced melanoma.

Animal venoms have emerged as a promising source of bioactive compounds with diverse pharmacological properties, including anticancer [4]. A striking example is the venom-derived peptide gomesin (AgGom), originally isolated from the hemocytes of the Brazilian spider *Acanthoscurria gomesiana* [5]. It is an 18-amino acid peptide, consisting of a β-hairpin structure stabilized by two disulfide bonds [5, 6]. HiGom, an ortholog of AgGom derived from the venom gland of the spider *Hadronyche infensa*, shares similar structural features, including C-terminal amidation and disulfide connectivity [7]. Both peptides exhibit broad-spectrum antimicrobial activity and potent cytotoxic properties against various cancer cell types, including melanoma [7]. At low concentrations, HiGom is more potent than AgGom in diminishing the viability of *BRAF*-mutated melanoma cells, and with minimal impact on healthy cells at effective doses [7]. In *BRAF*-melanoma xenograft mouse and zebrafish models, both HiGom and AgGom significantly reduce tumor growth and melanoma cell proliferation [7].

Gomesin peptides exert their cytotoxic effects through a complex, multi-pronged mechanism that combines membrane disruption with well-orchestrated intracellular actions [8–10]. Membrane permeabilization is achieved by binding to the phospholipid bilayer component of cell membranes with a preference for negatively charged lipids such as phosphatidylserine (PS), a lipid enriched in cancer cell membranes [8]. The peptides show limited effect on healthy cells [7], which have lower PS levels and higher levels of neutral lipids such as phosphatidylcholine (PC) or cholesterol (Chol) [8]. Cholesterol is a vital lipid involved in various physiological and pathological processes, including the interaction and uptake of therapeutic peptides by cellular membranes [9]. Its presence has been shown to reduce the lytic activity of gomesins in giant unilamellar vesicles (GUVs) [10]. Likewise, increasing cholesterol levels in cells cause a decrease in the cytotoxicity of gomesins [11], indicating a protective cell mechanism.

The unique lipid composition of melanoma cell membranes may influence the selective cytotoxicity of gomesins particularly in *BRAFV600E* cells. Previously, we demonstrated that gomesin peptides target melanoma *BRAFV600E* cells [7]. In this study, we investigated how the lipid composition mediates the activity and internalization of these peptides, identified key cytotoxic targets using CRISPR/Cas9 screening, and validated the findings in vitro and in vivo. Overall, this study provides valuable new insights into the role lipids play in promoting the cytotoxicity of gomesins, leading to new targets and strategies in developing the next generation of targeted melanoma therapies.

## Results

### The specific anti-melanoma properties of gomesin peptides

**Fig. 1:**
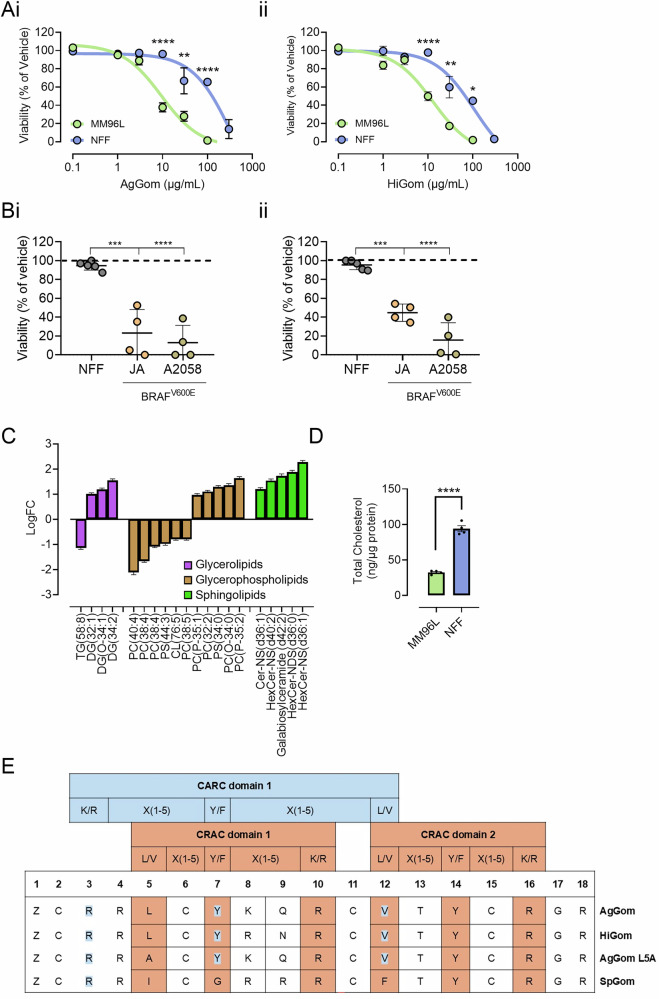
The effects of AgGom and HiGom on cell viability, lipid composition, and cholesterol levels in melanoma cells and fibroblasts. **A** Concentration response curve for the effects of a 48 h treatment with (**i**) AgGom and (**ii**) HiGom on the viability of MM96L cells and NFF cells (*n* = 3). **B** Scatter plots showing the effect of (**i**) AgGom (10 μg/mL, 48 h) and (**ii**) HiGom (10 μg/mL, 48 h) on the viability of NFF in the indicated *BRAFV600E* melanoma cells (*n* = 4–5). **C** Classification of the 20 most significantly altered lipids identified in the lipidomic screening, where 55% were glycerophospholipids (purple), 20% glycerolipids (brown), and 25% as sphingolipids (green). **D** Bar-chart of the total cholesterol content in MM96L and NFF cells analyzed spectrophotometrically (*n* = 4). **E** Identification of the Cholesterol Recognition Aminoacid Consensus sequence (CRAC) or its reverse form (CARC) in AgGom, HiGom, AgGom L5A and SpGom peptides. Data are shown as mean ± SEM. Statistical significance was assessed by one-way ANOVA test with Šídák’s correction in panels A and B and with two-sided unpaired Student’s t test in panel D (*p < 0.05, **p < 0.01, ***p < 0.001 and ****p < 0.0001 vs control cells).

AgGom and HiGom (Fig. S1 and Table S1) are spider-derived peptides with various documented activities, including antimicrobial and antitumoral [7, 8, 12]. The antimelanoma properties of gomesin peptides have been of special focus in our lab [7] and in others [13]. Comparison of the concentration-dependent reduction in the viability of human melanoma (MM96L) cells and healthy fibroblasts (NFF) demonstrated a preferred antitumoral effect for both AgGom (IC_50_: 9.5 µg/mL in MM96L vs. 71 µg/mL in NFF) and HiGom (IC_50_: 9.8 µg/mL in MM96L vs. 59 µg/mL in NFF). Specifically, the peptides had a potency 6-7 times higher in melanoma cells than in fibroblasts (Fig. 1Ai, Aii**)**. AgGom and HiGom exerted a reduction in proliferation across a range of *BRAF*^*V600E*^ melanoma cell lines (Fig. 1Bi, Bii**)**. Thus, we further investigated the mechanisms by which gomesins target melanoma cells using various cell death inhibitors **(**Fig. S2A**)**, including necrostatin-1 (necroptosis inhibitor), ferrostatin-1 (ferroptosis inhibitor), VX-765 (caspase 1/4 inhibitor, pyroptosis) and z-VAD FMK (pan-caspase inhibitor). Interestingly, our results suggest that gomesins may not act through a single, well-characterized cell death pathway. Instead, they appear to trigger a combination of different cell death pathways or engage alternative, less well-understood mechanisms. These data highlight the diverse ways these peptides can affect cell viability.

### Lipid composition in melanoma could potentially explain differences in gomesin potency

Gomesin peptides have distinct membrane affinities and disrupting-activities depending on the lipid composition in model membranes [8, 12]. Therefore, we explored possible discrepancies in the lipid composition of melanoma cells and fibroblasts that might mediate binding affinity and, consequently, the bioactivity of gomesins in melanoma cells. We reported a total of 1703 lipids via lipidomics analysis. Lipidomic screening using a robust linear model estimation revealed a distinct subset of 709 lipids (Fig. S2B). Of those, the content of 463 lipids (~65%) was significantly altered between fibroblasts and melanoma cells. Among the 20 lipids displaying the lowest adjusted *p*-values, 55% belonged to the glycerophospholipid class, 20% were glycerolipids and 25% were sphingolipids (Fig. 1C). Intriguingly, all reported sphingolipids were elevated in melanoma cells, with 67% (4 out of 6) being neutral glycosphingolipids. It is essential to emphasize that melanoma cells consistently displayed lower levels of unsaturated glycerophospholipids (PC 40:4, PC 38:4, PC 38:5, PS 44:3) and triglycerides (TG 58:8), which may imply a lower fluidity in these cells than fibroblasts. In that regard, cholesterol is an integral component in specialized microdomains that regulates cell signaling and influences membrane fluidity [9]. Indeed, cholesterol interacts with sphingolipid molecules and generates liquid-ordered microdomains known as lipid rafts [9]. Spectrophotometric examination revealed that melanoma cells contain approximately three times lower total levels of cholesterol than fibroblasts (Fig. 1D). Compellingly, an inspection of the peptides’ sequences using the Fuzzpro application in the EMBOSS suite (https://www.bioinformatics.nl/cgi-bin/emboss/fuzzpro), highlighted the presence of several potential cholesterol-binding motifs for HiGom and AgGom: two well-defined cholesterol recognition/interaction amino acid consensus or CRAC regions [14] (amino acids 5–10 and 12–16/18) and one reverse CARC domain (sequence 3/4-12) (Fig. 1E**)**. The CRAC motif is characterized by the sequence pattern of L/V-X(1–5)-Y/F-X(1–5)-R/K and is commonly located in the inner leaflet of the plasma membrane. Conversely, the CARC motif follows the sequence R/K-X(1–5)-Y/F-X(1–5)-L/V generally found in the outer leaflet of the plasma membrane [15]. As proof of concept for the importance of CRAC domains, substituting L5A in AgGom (AgGomLA5) (Fig. S1 and Table 1) eliminated one potential CRAC domain, which correlated with reduced cytotoxicity (Fig. S2C). It is noteworthy, that the variant SpGom (Fig. S1 and Table 1), which presented the lowest cytotoxicity in MM96L cells and fibroblasts at the concentrations tested (Fig. S2C) failed to display any potential cholesterol binding site (Fig. 1E).

**Fig. 2:**
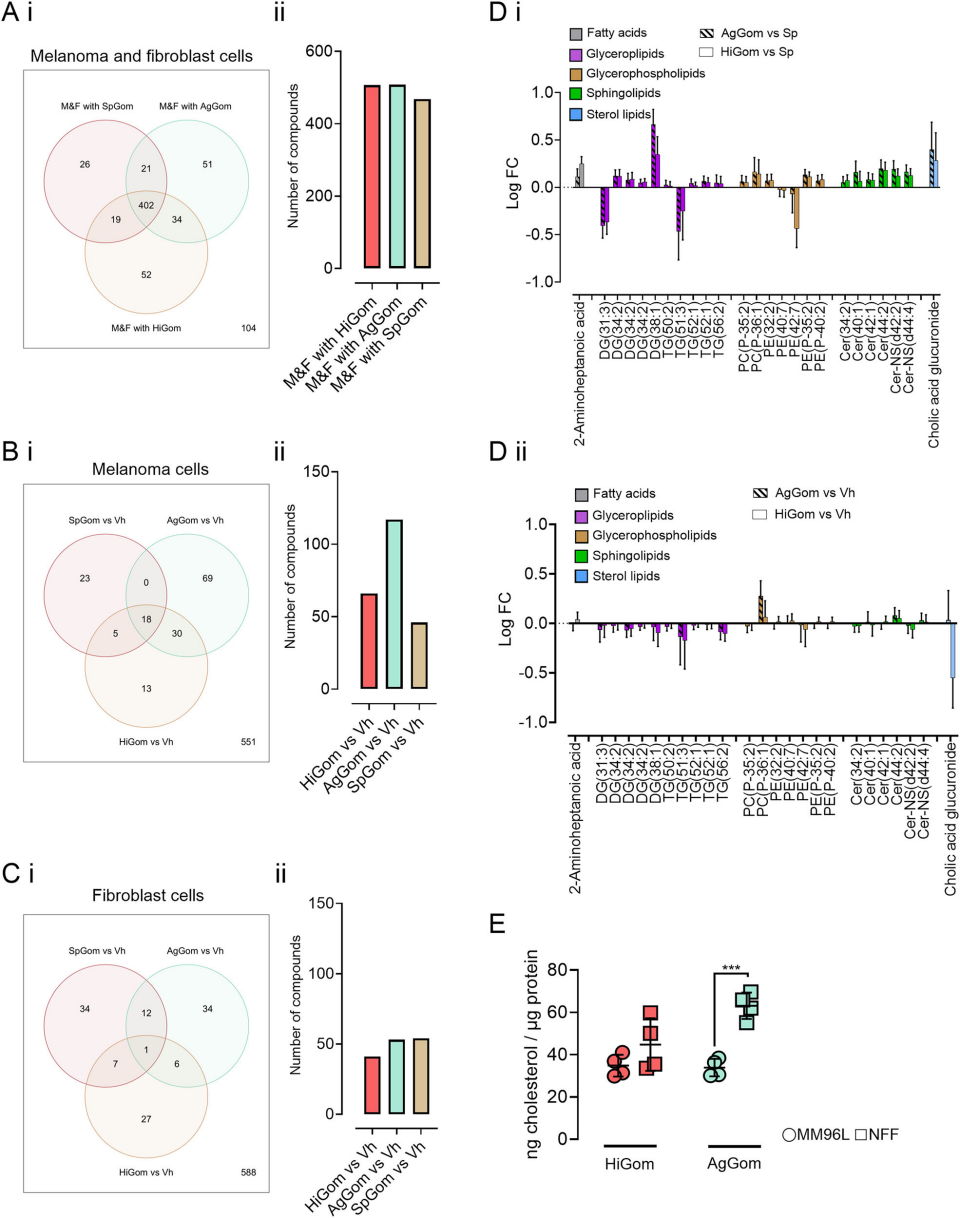
Comparative lipidomic analyses higlights differential lipid expression patterns and cholesterol levels in melanoma and fibroblast cells following treatment with gomesin peptides. Venn diagrams illustrate the overlap in differentially expressed lipids between **Ai** melanoma cells and fibroblasts treated with HiGom, AgGom or SpGom, and differentially expressed lipids between **Bi** melanoma cells or **Ci** fibroblasts treated with HiGom, AgGom or SpGom versus vehicle (Vh). Graph bars (**Aii, Bii, Cii**) represent a quantification of the lipids overlaps and differences visualized in Venn diagrams. **D** Distribution of differentially expressed lipids (grouped as fatty acids, glycerolipids, glycerophospholipids, sphingolipids and sterol lipids) in (**i**) melanoma and (**ii**) fibroblasts samples under AgGom and HiGom treatments vs vehicle. Lipids are plotted based on their LogFC, showing an overexpression (above 0) or underexpression (below 0). **E** Quantification of total cholesterol levels in MM96L and NFF cells after HiGom (red) and AgGom (green) treatments (*n* = 4). Statistical significance was assessed by a one-way ANOVA test with Šídák’s correction (***p < 0.001 vs control cells).

We then repeated lipidomics experiments following treatment with AgGom and HiGom, as compared to SpGom and the vehicle controls in melanoma cells and fibroblasts. First, we analyzed distinct lipids grouped for AgGom, HiGom or SpGom treatments, irrespectively of cell type (i.e., MM96L and NFF). This analysis revealed that both melanoma and fibroblasts shared 468 differential lipids following treatment with the least cytotoxic peptide SpGom (Fig. 2A). By contrast, the cytotoxic AgGom and HiGom peptides presented 508 and 507 differential lipids, respectively (Fig. 2A). Based on this observation, we focused our investigation on 34 lipids that were consistently altered in both cell lines treated with the active peptides AgGom or HiGom (Fig. 2Ai). These shared deregulated lipids encompassed ~6% fatty acids (2 out of 34), ~23% glycolipids and sphingolipids (8 out of 34), and ~32% glycerophospholipids (11 out of 34).

We observed distinct lipid profiles in the treated groups compared to the vehicle controls in both cell lines separately (Fig. 2B, C). Specifically, for the MM96L group, there were 117 and 66 different lipids in the comparisons of AgGom vs. vehicle and HiGom vs. vehicle, respectively (Fig. 2B). Notably, similar comparisons in NFF revealed fewer differential lipids (Fig. 2C), with 53 lipids in the comparisons of AgGom vs. vehicle and 41 lipids in the HiGom vs. vehicle group. This suggests that the mediation of HiGom or AgGom on lipids is consistently more profound in melanoma cells than in fibroblasts (Fig. 2D). By contrast, the number of altered lipids in the comparison of SpGom vs. vehicle were higher in NFF than in MM96L (Fig. 2B, C). Moreover, fibroblasts displayed only six common differential lipids in response to both AgGom and HiGom treatments (Fig. 2Ci). By contrast, melanoma cells exhibited 30 shared differential lipids following exposure to AgGom or HiGom (Fig. 2Bi). Consequently, we conducted an in-depth examination of these 30 candidates to gain a deeper insight into the impact of AgGom and HiGom on the lipid composition in melanoma cells. We categorized the affected lipids into three primary categories, namely glycosphingolipids (comprising 10 out of the 30 lipids, or 33,3%), glycerophospholipids (7 out of 30 lipids, or 23,3%), and sphingolipids (6 out of 30 lipids, or 20%) (Fig. 2Di). Alterations were observed in fatty acids and sterols, each accounting for just 1 out of the 30 lipids (3,3%). The changes detected in the lipids of fibroblasts treated with AgGom or HiGom compared to the vehicle were much smaller (Fig. 2Dii) than those observed in melanoma.

Notably, we identified that AgGom and HiGom consistently reduced polyunsaturated lipids (>3 double bonds) such as glycerolipids and glycerophospholipids (Fig. 2Di). This suggests that the membrane fluidity in cells may be jeopardized. Therefore, we further investigated the total cholesterol content as another key element of membrane fluidity. We noticed that HiGom and AgGom, reduced the total cholesterol content in fibroblasts (Fig. 2E). Specifically, HiGom diminished it to a level comparable to that observed in melanoma cells. This implies that AgGom and HiGom could lessen total cholesterol levels in cells with high content, such as the studied fibroblasts.

We propose that the identified lipids may serve as potential biomarkers for the cytotoxicity of AgGom and HiGom in melanoma. This is further supported by their presence in comparisons of AgGom or HiGom with the less cytotoxic SpGom (Fig. 2).

### Phosphatidylserine and cholesterol modulate gomesin binding and disruption differently in model membranes and cellular models

Cholesterol, a key constituent of mammalian membranes, influences membrane properties and modulates gomesin activity [8]. In addition, the interaction of gomesins with negatively charged lipids, such as PS enhances the membrane-disrupting activity of cholesterol in model membranes [8, 12]. It is worth noting that PS is generally found in the inner leaflets of mammalian cell membranes [16]. We used a series of biophysical experiments to understand the effect of cholesterol and PS on the membrane-altering activity of AgGom and HiGom in model membranes as compared to cellular models.

**Fig. 3:**
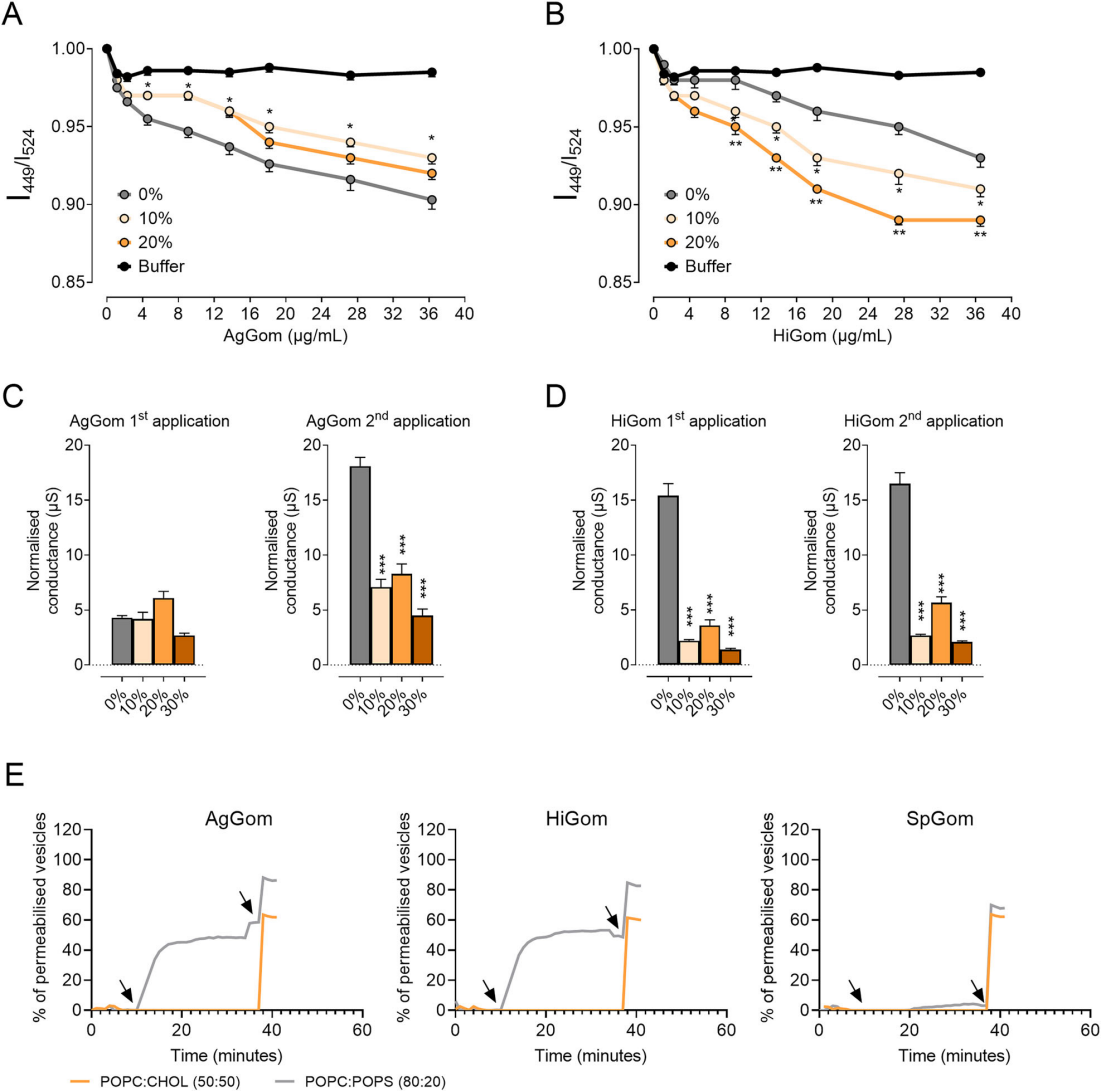
Cholesterol mediates the effect of gomesin peptides on membrane binding, disruption, and permeability. Ratio of the RH421 fluorescence intensity for increasing concentrations of **A** AgGom or **B** HiGom in model membranes (4 POPC: 1 POPS) with increasing % of cholesterol (*n* = 5–9). Electrochemical impedance spectroscopy determines membrane disruption induced by 10 μg/mL of **C** AgGom or **D** HiGom and the effects of acute (1st application) or cumulative (2nd application) (*n* = 5–9). **E** Curves represent calcein release activity (*n* = 3) exerted by AgGom, HiGom, and SpGom with POPC:CHOL 50:50 (orange) and POPC:POPS 80:20 (grey) lipid model vesicles over time. The first arrow indicates the addition of the peptides (2 mg/ml) and second arrow the addition of detergent to lysate vesicles. Statistical significance was assessed by one-way ANOVA test with Šídák’s correction (*p < 0.05 **p < 0.01 and ***p < 0.001 vs control conditions).

We used RH421 fluorescence spectra, based on the ratio of intensity at two specific wavelengths, to estimate the effect of cholesterol on the binding of AgGom and HiGom to model membranes with a constant 4:1 POPC:POPS ratio and increasing cholesterol concentrations. The RH421 spectra indicate that increasing cholesterol content inversely affects AgGom binding to model membranes, while HiGom binding to the membrane is favored by increasing % of cholesterol (Fig. 3A, B). We also investigated the effect of cholesterol on membrane disruption by electrochemical impedance spectroscopy (EIS) on tethered bilayer lipid membranes, which were treated with 10 µg/mL AgGom or HiGom. The effects were studied after acute (first application) or accumulative (second application) and membrane disruption was reported as normalized conductance (Fig. 3C, D). The EIS data revealed accumulative membrane disruption following sequential applications of AgGom but not for HiGom, where disruption was high and saturated after the first application. Independent of these differences, membrane disruption was reduced for both peptides with increasing levels of cholesterol. This implied that cholesterol had a protective effect against membrane disruption regardless of whether it increased or decreased membrane binding.

The role of lipids in facilitating gomesins’ ability to permeabilize the membranes was further confirmed using the calcein leakage assay. The membrane-disruptive activities of AgGom, HiGom, and SpGom peptides were assessed in three distinct lipid model vesicle compositions: POPC:CHOL (50:50) and POPC:POPS (80:20). AgGom and HiGom displayed no activity in POPC:CHOL (50:50) membrane composition but in POPC:POPS (80:20) revealed high permeabilization (Fig. 3E). As expected, SpGom had no activity at all tested vesicle compositions. Overall, these results emphasized that both AgGom and HiGom possessed membrane-disruptive capabilities mediated by lipid compositions enriched with phosphatidylserine.

In summary, our experiments with model membranes and cells revealed that cholesterol modulates gomesin activity by reducing membrane disruption, despite its varying effects on peptide binding. While gomesins strongly permeabilized phosphatidylserine-rich membranes, they were ineffective in cholesterol-rich membranes, highlighting the crucial role of lipid composition in their cytotoxicity.

**Fig. 4:**
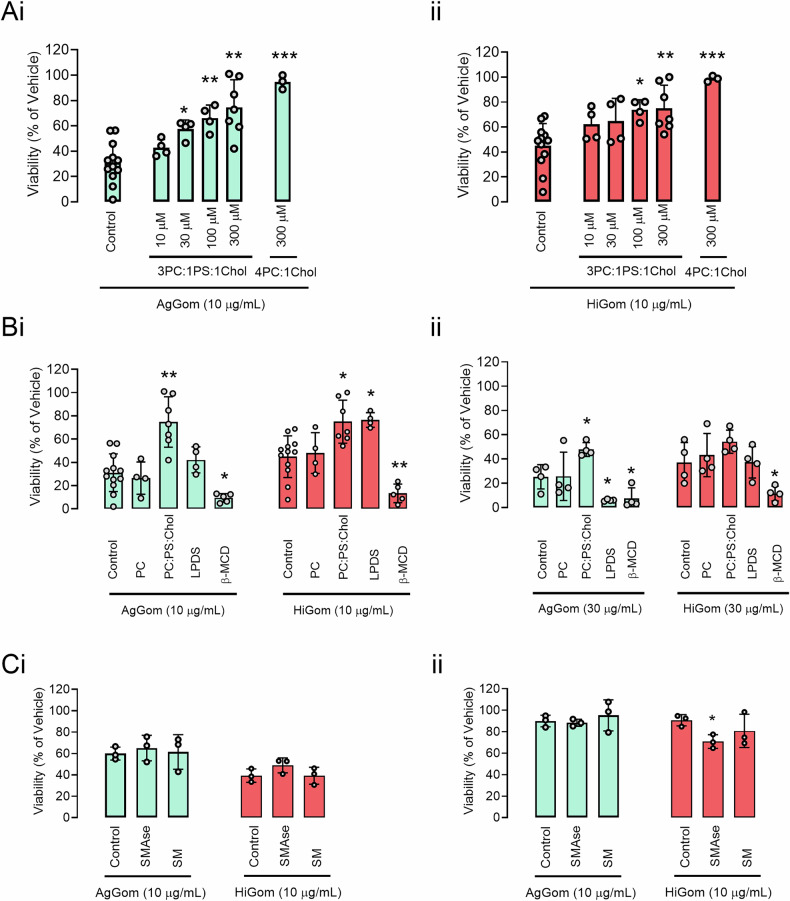
**Lipids and cholesterol supplementation or depletion influence the cytotoxicity of gomesin peptides on melanoma cells and fibroblasts. A** Effects of a 48 h treatment with 10 μg/mL (**i**) AgGom or (**ii**) HiGom in the viability of MM96L cells under control conditions or supplemented with increasing concentrations of PC:PS: Cholesterol at a ratio 3:1:1 or PC: Cholesterol at a ratio 4:1 (*n* = 3–8). **B** Effects of a 48 h treatment with AgGom or HiGom in the viability of (**i**) MM96L and (**ii**) NFF cells under control conditions or supplemented with 300 µM of the indicated lipids (PC, 3PC:1PS:1Chol), β-MCD at 1 mM or 10% of lipoprotein-deficient serum (LPDS) (*n* = 3-8). **C** Effects of a 48 h treatment with AgGom or HiGom in the viability of (**i**) MM96L and (**ii**) NFF cells under control conditions or supplemented with SMASE or SM at 100 μM (*n* = 3). Data are shown as mean ± SEM. Statistical significance was assessed by one-way ANOVA test with Šídák’s correction (*p < 0.05 **p < 0.01 and ***p < 0.001 vs control conditions).

These data complemented our cell-based experiments, where the addition of cholesterol in media with a defined (3PC:1PS) composition significantly diminished the melanoma viability for both gomesin peptides (Fig. 4A**)** compared to media containing PC lipids only. The addition of lipoprotein deficient serum (LPDS) also reduced the cytotoxicity mainly of HiGom, while β-MCD, which sequesters cholesterol and disrupts lipid rafts microdomains at the plasma membrane [17, 18], enhanced the cytotoxicity for both peptides (Fig. 4B). Interestingly, altering SM levels, whether through supplementation or deprivation, had no effect on the properties of gomesin peptides in MM96L. However, supplementation with sphingomyelin phosphodiesterase (SMase) in NFF increased the antiproliferative capacity of HiGom **(**Fig. 4C**)**.

### Identification of key cytotoxic genes of gomesin using CRISPR/Cas9

**Fig. 5:**
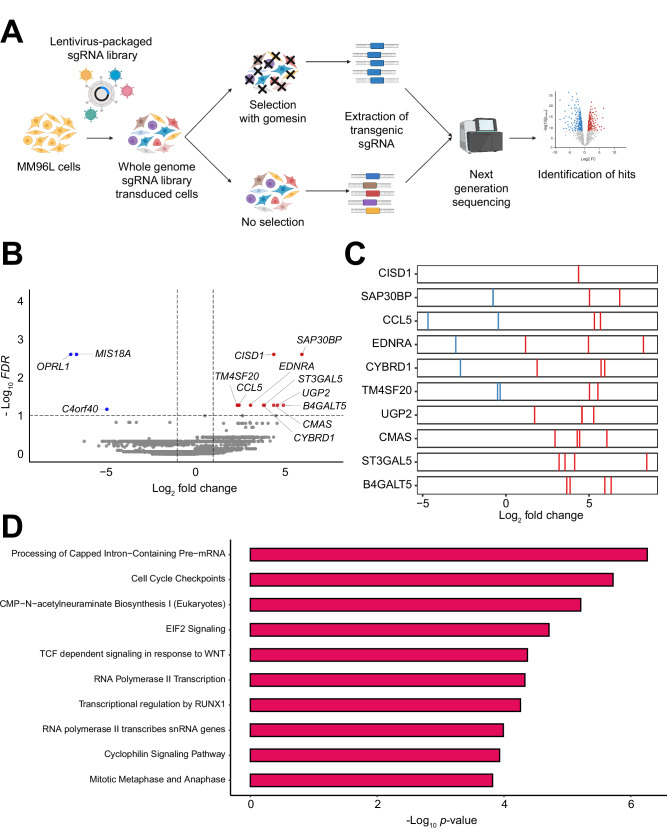
**A genome-wide CRISPR knockout screen reveals genes and pathways that modulate cellular sensitivity to gomesin peptides in melanoma cells. A** Schematic of workflow for gomesin whole genome CRISPR knockout (KO) screen. MM96L cells were transduced at MOI = 0.3 with lentivirus packaging the Toronto Knockout sgRNA library (TKOv3). Four rounds of selection with 25 µM gomesin (LD90) were performed followed by genomic DNA extraction, next-generation sequencing and gene enrichment analysis. Schematic made using Biorender. **B** Gene enrichment analysis performed by comparing read count abundance of sgRNA in selected and non-selected samples using MAGeCK with control sgRNA normalization. Horizontal dotted line indicates FDR = 0.1. Vertical dotted lines indicate log2 fold change (LFC) of -1 and 1. Results visualized using MAGeCKFlute and enhancedvolcano R packages. **C** LFCs of individual sgRNA for the top 10 enriched genes by FDR. **D** Top 10 Ingenuity Canonical Pathways identified using Ingenuity Pathway Analysis with positive selection *p*-value < 0.05 and absolute value of positive selection LFC > 1.

To further investigate the molecular drivers of gomesin cytotoxicity in melanoma cells, we used CRISPR/Cas9 genetic editing in MM96L cells (Fig. 5A). This allowed us to identify key genes and pathways involved in the response to HiGom, a representative gomesin peptide. In brief, wildtype MM96L melanoma cells were transduced at a multiplicity of infection (MOI) of 0.3 with lentivirus packaging the whole genome Toronto Knockout sgRNA Library (TKOv3). This library targets 18,053 genes with four independent sgRNAs per gene, maximizing the likelihood of a single sgRNA per cell. The transduced cells were then selected using HiGom (approx. LD90 concentration). Four rounds of selection were performed, where each round was defined as 72 h treatment with HiGom followed by 72 h recovery in regular culture media. Gene enrichment analysis was performed by comparison of read count abundance between selected samples and unselected diversity controls using MAGeCK. Our knockout (KO) screen identified 13 hits (based on FDR < 0.1 and log_2_ fold change < −1 or > 1): 10 enriched genes (i.e., KO of these genes confer resistance to HiGom) and three depleted genes (KO of these genes promote sensitivity to HiGom) (Fig. 5B, C). Notably, several of these hits encoded membrane proteins, including the enriched EDNRA15 [19] and TM4SF20, as well as the depleted hit OPRL1. Additionally, two hit genes, ST3GAL5 and B4GALT5, were linked to sphingolipid metabolism [12, 20].

Ingenuity Pathway Analysis (IPA) was conducted using only genes with positive selection *p*-value < 0.05 and positive selection absolute log_2_ fold change greater than 1 (903 genes, Fig. 5D**)**. Notable amongst the top canonical pathways were those involved in the biosynthesis or metabolism of polysaccharides found in glycoprotein chains (CMP-N-acetylneuraminate biosynthesis I (Eukaryotes); GDP-mannose biosynthesis; sialic acid metabolism; and colonic acid building blocks biosynthesis).

### Mechanistic validation of the individual key effector-genes for gomesin cytotoxic

**Fig. 6:**
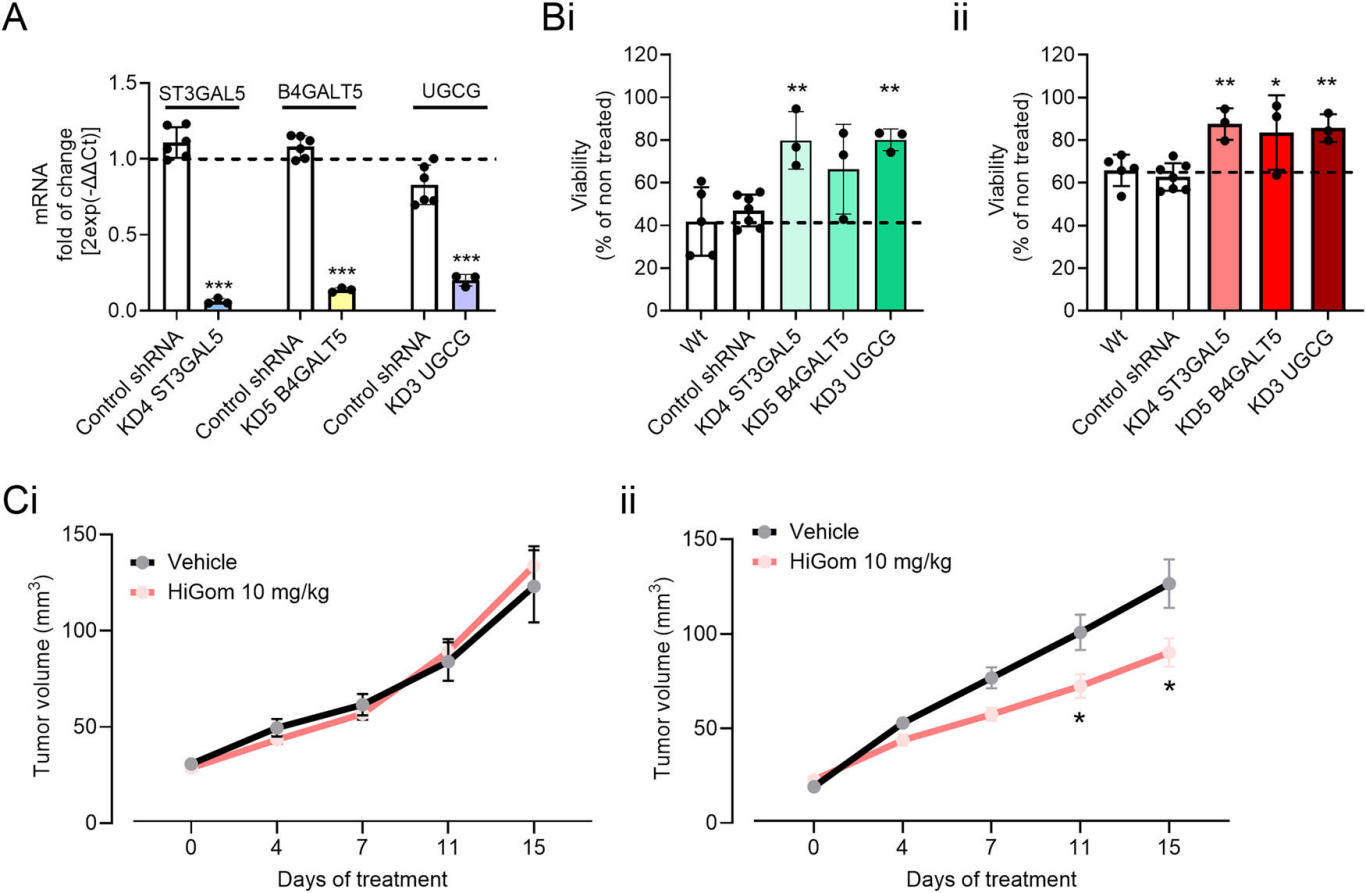
**Validation of the role of glycosphingolipid-related genes in gomesin peptide sensitivity through gene knockdown and in vivo melanoma models. A** Validation of gene silencing at the RNA level for ST3GAL5, B4GAL5, and UGCG using qRT-PCR. Cells transduced with silencing shRNAs compared to control cells transduced with non-targeting shRNA (white bars). Results are presented as mean ± standard error (*n* = 6); statistical significance was determined using a two-sided unpaired t-test. Effect of **Bi** AgGom and **Bii** HiGom on cell viability measured using the MTT assay. Non-transduced cells (white bars), control-transduced cells (white bars), and knockdown cells (green and red bars) were treated with the peptides at 10 μg/mL for 48 hours. Results are shown as mean ± standard error (*n* = 3), and statistical significance was analyzed using one-way ANOVA. **Ci** ST3GAL5 knockdown MM96L shRNA and **Cii** MM96L control melanoma xenograft mouse models tumor progression under vehicle (black) or HiGom (10 mg/Kg) (red) treatment for 15 days. Tumor volume measurements are presented as mean ± standard error (*n* = 5 mice, 10 tumors). Data are shown as mean ± SEM. Statistical significance was assessed by one-way ANOVA test with Dunnett’s correction in panels A and B, and two-way ANOVA test with Šídák’s correction for panel C vs vehicle (*p < 0.05 **p < 0.01 and ***p < 0.001 vs control conditions).

In the IPA, pathways related to glycosphingolipid biosynthesis were enriched, pointing to the importance of lipid composition in the cellular response to HiGom. *ST3GAL5* and *B4GALT5* are key enzymes in glycosphingolipid metabolism, suggesting that disruptions in these pathways could affect HiGom uptake or its cytotoxic effects. Similarly, *UGCG*, involved in regulating the balance of ceramides and glucosylceramides, further emphasized the potential impact of lipid metabolism on drug sensitivity. These genes were chosen for validation because they directly link lipid biosynthesis to melanoma cell response, making them strong candidates for further investigation. When we analyzed the expression of these genes, we observed that they were downregulated in fibroblasts compared to melanoma cells (Fig. S3), which could explain the cytotoxic discrepancies of gomesin peptides. Therefore, we followed up by using shRNA knock-down in melanoma cells to target the hits identified from our CRISPR KO screen (*ST3GAL5*, *B4GALT5, UGCG, p* < 0.05*, FDR* < 0.2). We selected the best candidate from a battery of shRNAs for each of these genes (Fig. 6A) and confirmed through viability assay that individually knocking down *ST3GAL5*, *B4GALT5* or *UGCG* diminished the cytotoxicity of both AgGom and HiGom in melanoma cells (Fig. 6B). The loss of cytotoxicity of gomesins in cells expressing these shRNAs indicated that these genes, or the pathways they were part of, are involved in the targeted antitumor mechanism.

### In vivo validation of ST3GAL5 as the representative gene of the glycosphingolipid pathway driving cytotoxicity in gomesins

To confirm the physiological relevancy of these hits and glycosphingolipids in the cytotoxicity of gomesins in vivo, we selected the *ST3GAL5* as the representative gene for the glycosphingolipid pathway. First, we generated *ST3GAL5*-deficient MM96L xenograft melanoma tumors in nude mice and assessed whether HiGom maintained its antitumorigenic properties. Accordingly with our in vitro experiments using shRNA *ST3GAL5* KD MM96L cells, HiGom-treated *ST3GAL5*-deficient xenograft tumors exhibited the same growth as the *ST3GAL5*-deficient xenograft tumors from the vehicle-treated mice (Fig. 6C i). By contrast, mice with wild type (WT) MM96L xenograft tumors treated with HiGom displayed significant tumor regression (*p* value = 0,0296 at day 11) in comparison to the vehicle-treated group (Fig. 6C ii).

All together, these experiments confirmed the importance of the glycosphingolipid pathway in the antitumoral properties of gomesin peptides against melanoma.

## Discussion

This study provided compelling new evidence that the anti-tumoral properties of AgGom and HiGom effectively target melanoma (MM96L) cells while having minimal impact on fibroblasts (NFF). This aligned with previous studies on the specific anticancer properties of gomesin peptides, either derived from natural sources (such as spider hemocytes or venom transcriptome) or chemically modified in labs [7, 12]. Cytotoxicity is influenced by differences in lipid composition between cancerous and healthy cells.

Gomesins interact with the negatively charged phospholipids in cancer cell membranes, creating pores that disrupt the membrane and lead to cell death via the hijack of signaling pathways such as MAPK or PI3K [7, 21], triggering apoptosis through reactive oxygen species (ROS) generation, calcium overload, and mitochondrial dysfunction [7, 22].

The distinct activities of AgGom and HiGom across different lipid profiles highlighted the intricate relationship between lipid composition and peptide sequence in determining membrane-disrupting and cytotoxic effects. The lack of membrane-disrupting activity for both peptides in the POPC:CHOL (50:50) membrane, as indicated by dye leakage assays, suggested that these effects may depend on the presence of specific lipid species, such as phosphatidylserine or sphingomyelin. This conclusion was supported by experiments in model membranes incorporating lipids such as POPC:POPS (80:20) further highlighting their preference for negatively charged lipid environments [8].

Our findings feature the cholesterol-dependent interactions of AgGom and HiGom, offering further insight into their antitumoral properties. AgGom had reduced binding in cholesterol-rich membranes, while HiGom exhibited enhanced binding under the same conditions. These suggested distinct mechanisms of membrane interaction, potentially involving cholesterol-specific binding motifs or lipid packing effects. Additionally, the cumulative membrane disruption observed with AgGom indicated that this peptide might uniquely induce progressive membrane destabilization over time, likely through sustained interactions with lipid bilayers.

In artificial membranes (POPC:POPS, 4:1), both AgGom and HiGom bound to and disrupted membranes. HiGom achieved maximum disruption with a single application, whereas AgGom required a second application to reach a comparable effect. Despite these differences, the presence of cholesterol reduced the disruptive capacity of both peptides, with HiGom being particularly affected.

Interestingly, melanoma cells, which rely heavily on the metabolism of fatty acids for the generation of energy, were more susceptible to the treatment with AgGom or HiGom than NFF cells and induced more pronounced changes in lipid composition, suggesting a differential impact on lipid metabolism. Metabolic reprogramming, including a switch from glycolysis to lipid metabolism, plays a crucial role in melanoma progression and treatment resistance, making lipid-targeting therapies such as gomesins potentially promising drug candidates [23, 24].

Cholesterol has been associated with cancer progression, but its role remains complex [25]. In our study, adding cholesterol or its precursor (desmosterol, data not shown) to melanoma cells decreased the cytotoxicity of gomesins, suggesting a chemotherapy-resistance mechanism. Melanoma cells furthermore exhibited lower cholesterol levels when compared to fibroblasts, which could explain their high cytotoxic sensitivity for gomesins. The ability of gomesins to bind cholesterol, as shown by their CRAC domain, may restrict melanoma cell proliferation. However, the association and interplay among drug action, cholesterol, and cancer progression are complex and most likely tumor-specific, mediated by genetic and metabolic factors that warrant further investigations in future studies.

Accordingly with these results, membrane permeability and disruption experiments using cholesterol-removing β-MCD, boosted the cytotoxicity of gomesins in melanoma cells. However, only AgGom achieved compatible results in fibroblasts. When LPDS was added, the cytotoxicity of HiGom decreased in melanoma cells, but increased in fibroblasts, highlighting the multifaceted role of phospholipids and cholesterol in cell-specific manner responses.

Our lipidomics analysis revealed that gomesin-induced cytotoxicity correlated with a melanoma-specific lipid fingerprint, characterized by a sustained alteration in the lipidic cellular landscape, resulting in more than 60% difference compared to fibroblasts. The primary lipid classes affected by gomesins in melanoma cells included glycerophospholipids, glycerolipids, and sphingolipids. Notably, melanoma cells exhibited higher levels of saturated glycerophospholipids, whereas fibroblasts had increased levels of unsaturated glycerophospholipids and triglycerides. These findings underscore the critical role of lipid metabolism in driving the cytotoxic effects of gomesins.

The significance of our findings was reinforced by the identification of key drivers of gomesin cytotoxicity linked to glycosphingolipid synthesis, a hallmark of melanoma-specific lipid composition. Glycosphingolipids are essential for membrane integrity and play a critical role in cancer progression [26]. Our loss-of-function experiments, including CRISPR/Cas9 genome-wide editing and transient shRNA-mediated knockdown of glycosphingolipid-related genes, along with in vivo studies on HiGom treatment in *ST3GAL5*-deficient xenograft melanoma models, confirmed that glycosphingolipid biosynthesis was crucial for the efficacy of gomesin.

In summary, this study provided a detailed characterization of the cellular environment required to optimize gomesin cytotoxicity against melanoma. Furthermore, our findings lay the groundwork for developing new strategies to enhance the antitumoral properties of gomesins. These strategies include lipid-enriched nutritional interventions (e.g., diets rich in glycosphingolipids and saturated fatty acids with reduced cholesterol) and synergistic therapies combining AgGom or HiGom with FDA-approved LXRα agonists, which promote cholesterol secretion and depletion. These approaches could lead to more effective melanoma treatments.

## Materials and methods

### Abbreviations

ACN–acetonitrile; calc.–calculated; DCM–dichloromethane; DIPEA–*N*-Ethyl-*N*-(propan-2-yl)propan-2-amine; DMF–dimethylformamide; eq.–molar equivalent; ESI-MS–electrospray ionization mass spectrometry; FA–formic acid; Fmoc–(9*H*-Fluoren-9-yl)methyl carbonochloridate; HCTU–*O*-(1*H*-6-Chlorobenzotriazole-1-yl)-1,1,3,3-tetramethyluronium hexafluorophosphate; MeOH–methanol; obs.–observed; RP-HPLC–reversed-phase high-performance liquid chromatography; SPPS–solid-phase peptide synthesis; TFA–trifluoroacetic acid; TIPS–triisopropylsilane; LPDS-lipoprotein deficient serum; MCD- methyl beta-cyclodextrin, tbML – thethered bilayer lipid membrane, EIS – electrical impedance spectroscopy.

### Analytical reversed-phase high-performance liquid chromatography (RP-HPLC) for gomesin peptides characterization

Peptides were analyzed using a C_18_ column (Kromasil 300-5-C18 4.6 × 50 mm) on a Dionex Ultimate 3000 system with UV detection at 214 nm (amide bond) and 280 nm (aromatics). Runs were performed using a linear gradient from 0-60% B in 60 min or 5–65% B in 30 min at a 1 mL/min flow rate. Solvent A: ddH_2_O + 0.1% TFA. Solvent B: ACN + 0.08% TFA.

### Mass spectrometry (MS) for gomesin peptide characterization

MS analysis was performed on a Thermo Scientific MSQ Plus ESI-MS in positive ion mode. Samples were injected at a flow rate of 1 mL/min (50% ACN/0.05% FA in water). High-resolution-MS analysis was performed on a Bruker maXis UHR-TOF (Qq-TOF) with ESI in positive ion mode. Samples were introduced *via* direct infusion in ACN/MeOH + 1% H_2_O at a 3 μL/min flow rate.

### Preparative RP-HPLC for gomesin peptides characterization

Peptides were purified using a C_18_ column (Kromasil 300-10-C18 250 × 21.2 mm) on a Waters AutoPurification HPLC system with UV detection at 214 nm. Purifications were performed using linear gradients 5–55% B in 60 min at a flow rate of 20 mL/min. Solvent A: ddH_2_O + 0.1% TFA. Solvent B: ACN + 0.08% TFA.

### Chemical synthesis of gomesin peptides

The nine peptides were assembled on a 0.1 mmol scale using a Symphony automated peptide synthesizer (Protein Technologies, Tucson, USA) and a Rink amide polystyrene resin (loading: 0.79 mmol/g) following standard Fmoc protocols. Fmoc amino acids were side-chain protected as Arg(Pbf), Asn(Trt), Cys(Trt), Gln(Trt), Lys(Boc), Thr(tBu) and Tyr(tBu). Couplings were performed in DMF using Fmoc-amino acid/HCTU/DIPEA (5 eq./5 eq./10 eq.; relative to resin loading) for 2 × 20 min. Fmoc deprotection was achieved using 30% (v/v) piperidine/DMF (2 × 2 min). After chain assembly and final Fmoc deprotection, the resin was washed with 50% MeOH in DCM and dried under vacuum. Peptide cleavage from the resin and removal of side-chain protecting groups were performed using TFA:H_2_O:TIPS (90:5:5) for 90 min at 25˚C. TFA was evaporated under N_2_ flow, and cold Et_2_O was added to precipitate the peptide. The precipitate was filtered, washed a second time with cold ether, dissolved in 0.05% TFA in 50% ACN/H_2_O, and lyophilized.

The purified peptides were oxidatively folded in 0.1 M NH_4_HCO_3_ buffer, pH 8.2, for 24 h at 25˚C using a peptide concentration of 100 μM. The folding was monitored by analytical RP-HPLC and ESI-MS, and the folded peptides were purified *via* preparative RP-HPLC. The folded gomesin analogs were of >95% purity, as determined by analytical RP-HPLC at 214 nm (wavelength of amide bond).

Information regarding the chemical structure, peptide sequence, analytical RP-HPLC chromatogram, and HRMS data for each gomesin peptide is provided in Fig. S1. In addition, a list of calculated and observed monoisotopic masses is provided in Table S1.

### Cell culture

MM96L melanoma cell line and the non-transformed NFF (control) cell line were maintained in a humidified incubator at 37 °C and 5% CO_2_ and in conditions as previously described [7, 27]. HEK293T cells were maintained in Dulbecco’s Modified Eagle’s Medium (DMEM; Thermo-Fisher), supplemented with 10% (v/v) Fetal Bovine Serum (FBS) and 0.1% penicillin-streptomycin (Gibco). MM96L cells (RRID: CVCL_D848) were maintained in Roswell Park Memorial Institute (RPMI) 1640 medium supplemented with 10% (v/v) FBS, 1% (v/v) Penicillin-Streptomycin, and 1% (v/v) GlutaMAX. All cells were cultured at 37 °C, 10% CO_2_, and atmospheric O_2_. All cell lines were mycoplasma-free.

### Cell viability assay

MTT assay (Sigma-Aldrich) was used to measure cell viability after 48 h of treatment with peptide, as previously described [7, 28, 29]. Absorbance was measured at 570 nm using a microplate reader (Victor Nivo; Perkin Elmer, Hamburg, Germany). For these assays, the effects of vehicle or treatments were expressed as the % of change of the non-treated cells or as % of the vehicle.

### Materials and reagents for model membranes

Tris(hydroxymethyl)-aminomethane (Tris, >99% purity), sodium chloride (>99.5% purity), and anhydrous ethanol were purchased from Sigma-Aldrich Australia. 1-palmitoyl-2-oleoyl-sn-glycero-3-phosphocholine (POPC, >99% purity), 1-palmitoyl-2-oleoyl-sn-glycero-3-phospho-L-serine (POPS, sodium salt, >99% purity) and cholesterol (>98% purity) were purchased from Avanti Polar Lipids Inc., through their Australian distributor Sigma-Aldrich Australia. All lipids were purchased as powder and dissolved in anhydrous ethanol at 3 mM. *N*-(4-Sulfobutyl)-4-(4-(*p*-(dipentylamino)phenyl)butadienyl)-pyridinium salt (RH421) was synthesized at the University of Sydney and kindly gifted by Dr Ronald Clarke. The dye was used without further purification. RH421 was dissolved in anhydrous methanol at a concentration of 1 μM. All buffers and peptide solutions were prepared using ultrapure water. Tris buffer was composed of 10 mM Tris 100 mM NaCl, and pH was adjusted to 7.0 $\pm$ 0.1 by dropwise addition of 3 M HCl. Peptide solutions were made by dissolving the peptide in tris buffer at the required concentration. If required, the pH of the peptide solution was adjusted to 7.0 $\pm$ 0.1 by dropwise addition of HCl or NaOH.

### Preparation of lipid vesicles for RH421 fluorescence measurements

LUVs with increasing cholesterol concentrations and a constant POPC:POPS ratio of 4:1 were prepared. The lipid compositions of the LUVs were 80% POPC – 20% POPS, 72% POPC – 18% POPS – 10% Chol, 64% POPC – 16% POPS – 20% Chol, 56% POPC – 14% POPS – 30% Chol. To prepare the LUVs, the required amount of lipid mixture was added to a glass vial, and the solvent evaporated off under a steady stream of nitrogen followed by keeping the vial in a desiccator under vacuum for 24 h. The lipid film was resuspended in tris buffer to achieve a lipid concentration of 300 µM. The sample was vortexed for at least 15 min, followed by 8 freeze/thaw cycles in a water bath of dry ice/acetone and warm water, respectively. Vesicles were then extruded with a 0.1 µm polycarbonate membrane, ensuring at least 20 passes through the membrane. The resulting LUVs were used on the same day.

### RH421 fluorescence measurements

Fluorescence spectra were collected with an Agilent Cary Eclipse fluorescence spectrometer. A quartz cuvette with a path length of 0.5 cm was used. Excitation scans from 250 nm to 650 nm were carried out with λ_em_ set to 670 nm (bandwidth 10 nm) with an RG645 filter (Schott) in front of the photomultiplier. The response time of the instrument was set to 0.1 s. Spectra were averaged over 5 scans.

For RH421 experiments, fluorescence spectra of LUVs with desired lipid composition in the presence of increasing concentrations of peptide were collected. First, a reference spectrum of LUVs with 0.5 μM RH421 and the absence of peptides was collected. For each subsequence spectra, the required amount of peptide stock solution (100 μM) was added to the cuvette to reach final peptide concentrations of 1.14, 2.28, 4.57, 9.13, 13.7, 18.26, 27.39 and 36.53 µg/ml, respectively. For each lipid composition and each peptide, at least four independent experiments were carried out. Control experiments were carried out for each lipid composition, in duplicate, using the same protocol using tris buffer instead of the peptide solution.

The raw spectra were used without background subtraction. From the spectra, the fluorescence intensities, *I*, at 455 nm and 524 nm were used to calculate the ratio I _455/570_ for each peptide concentration. Data in I _455/524_ vs. peptide concentration is reported as average ± standard error of the mean over at least four independent experiments. Spectra from buffer control experiments were analyzed separately, again, without background subtraction. Membranes were washed with 400 μL tris buffer every few minutes, at least three times, until a stable baseline was reached where stable was defined as at least 10 readings (frequency sweeps) where the absolute conductance did not change more than 0.1 μS after washing the membrane with buffer. Membranes were left for 10 min before addition of peptide solution to check for the absence of drift in conductance and capacitance readings. Statistical significance was evaluated using t-tests.

### Electrical impedance spectroscopy

Thethered bilayer lipid membranes were prepared using T10 gold-plated electrodes (catalog reference T10™, SDx Tethered Membranes Pty Ltd, Sydney, Australia) and equipment to form the tethered phospholipid bilayers were purchased from SDx Tethered Membranes, Pty Ltd, Sydney, Australia. TethaPod™ and tethaQuick™ for EIS were supplied by SDx Tethered Membranes, Pty Ltd, Sydney, Australia.

The impedance profiles were modeled in real-time using the tethaQuick™ software and a resistor-capacitor model. Excitation periods of 25 mV peak to peak were used. Frequencies from 0.1 Hz to 2 kHz were swept using four steps per decade, resulting in a sweep lasting between 70 to 80 seconds. Details of the AC impedance spectroscopy protocols used in this study can be found in Cranfield et al. [30] and Alghlayini et al. [31].

To achieve a stable baseline conductance, newly formed tBLMs were washed at least three times with 200 µL tris buffer. A stable baseline was defined as at least five readings with no more than 10% variation in absolute conductance. Membranes were then left for 10 min, during which conductance was monitored to ensure the absence of drift.

To measure the effect of AgGom and HiGom on the membrane permeability, the tBLMs were treated using the following protocol consisting of two cycles of addition and washout. For the addition, 200 µL of peptide at 4 µM (10 μg/mL) in tris buffer was added to the membrane. For the washout, 200 µL of tris buffer was added to membranes. After peptide treatment or washout, the membranes were left to equilibrate for at least 10 sweeps. For each lipid composition and peptide, at least five independent experiments on separate membranes were conducted. As a control, the same experiment was repeated using tris buffer only.

As the baseline conductance varies between individual tBLMs, the changes in membrane conductance upon the addition of peptide solution are reported as normalized conductance. Raw conductance was normalized using a dedicated baseline value for each membrane, which was calculated by averaging the conductance of the last 10 sweeps before the first addition of the peptide solution. The conductance after the first or second addition was calculated for each membrane by averaging the normalized conductance from the last five sweeps before the washout step. Normalized conductance values are reported as averages ± standard error of the mean over at least five independent experiments. Statistical significance was evaluated using t-tests.

### Lipid vesicles preparation for calcein assay

Lipids (Avanti Polar Lipids) were dissolved in chloroform and methanol in POPC:CHOL (50:50), POPC:POPS (80:20), and POPC:SM:POPS (64:20:16) ratios, and rotavoporated (Büchi) to form lipid films. The lipid solutions are dried out under a stream of nitrogen 2-3 h to ensure the evaporation of all the organic solvents. Warm hydration buffer (50 mM calcein, 1xPBS, pH 7.4) was added to warm up lipid films and vortexed intensely. Before extrusion, the MLVs were subjected to 6–10 freezing/thawing cycles with liquid nitrogen to improve encapsulation and samples were stored at -80 °C up to one month prior to large unilamellar vesicles (LUVs) formation. The MLVs suspension is passed through a 100 nm polypropylene filter (Whatman, Millipore) to ensure a homogeneous population of LUVs. Excess dye was removed by gel filtration through a Sepharose G-50 matrix.

### Release experiment

Vesicles (30 mM) and buffer (50 mM calcein, 1xPBS, pH 7,4) were pipetted to a black 96-well plate and measured at an excitation wavelength of 495 nm and with emission followed at 515 nm. To ensure non-spontaneous leakage, we recorded a fluorescence emission for 10 min prior to the addition of peptides. Gomesin peptides (2 mg/ml) were added, and fluorescence was measured for 20 min. Permeabilization was expressed as the percentage of maximal permeabilization obtained at the end of the assay by the addition of 0.1% Triton X-100.

### Whole genome CRISPR/Cas9 knockout (KO) screening

#### HiGom cytotoxicity titration

HiGom was resuspended in Dulbecco’s Phosphate Buffered Saline (DPBS; Sigma-Aldrich) MM96L cells were plated at a density of 1 × 10^4^ cells/well in 96-well plates. Titration was performed by incubating cells with 0–40 μM HiGom for 72 h. Cell viability was then determined by incubating cells in culture media containing 3 μg/mL resazurin sodium salt and incubating at 37 °C until a color change was observed. Fluorescence intensity was then determined using the FLUOstar Omega microplate spectrophotometer plate reader (544 nm excitation, 590 nm emission).

### Lentivirus production

HEK293T cells at approx. 80% confluence in T175 flasks were transfected with lentiviral packaging plasmids psPAX2 and pCAG-VSVG and the Toronto Knockout Library v3 (TKOv3; Addgene 90294) at a ratio of 3:1:3, respectively, using Lipofectamine 3000 Transfection Reagent (ThermoFisher Scientific) in Opti-MEM (Gibco) according to manufacturer’s instructions. A media change was performed approximately 16 h following transfection. Viral supernatant was then collected at 48 h post-transfection, filtered using 0.45 uM filters (Merck Millipore) and then concentrated via centrifugation at 3000 g using 100 K MWCO Pierce Protein Concentrators (Life Technologies Australia). The concentrated lentivirus was then aliquoted and stored at -80 °C until required.

Titration of TKOv3-containing lentivirus was performed by seeding MM96L cells at a density of 1 × 10^4^ cells per well in 24-well plates. ~24 h later, the MM96L cells were transduced with a TKOv3 dilution series in media containing 5 μg/mL Polybrene Infection/Transfection Reagent (Sigma-Aldrich). A media change was performed at approx. 16 h post-transduction to remove viral media, followed by a second media change that evening (approx. 24 h post-transduction) to administer 0.8 μg/mL Puromycin (ThermoFisher Scientific) to cells. After approx. 48 h of selection, viability was determined by cell count, and MOI was determined by comparison of the viability of transduced cells to that of non-transduced cells treated with puromycin and non-transduced cells that were not treated with puromycin.

### Whole genome CRISPR knockout screening

A cell suspension was generated containing 1 × 10^8^ MM96L cells, 5 μg/mL Polybrene Infection/Transfection Reagent (Sigma-Aldrich), and the volume of concentrated lentivirus previously determined to give a MOI of 0.3, such that the majority of transduced cells express a single sgRNA. This cell suspension was dispensed into four 6-well plates (2 mL per well) and spinoculation performed by spinning cells at 32 °C and 800 xg for 60 m. A media change was performed approx. 16 h later. At approximately 24 h post-spinoculation, the cells from each set of two plates were pooled to form two populations of TKOv3-transduced cells, referred to here as populations A and B. These cells were then seeded into T175 flasks in media containing 0.8 μg/mL puromycin. Puromycin selection was performed for two weeks, with passaging every 3–4 days. Following puromycin selection, each cell population was split into 2 × 5-layer T175 flasks (for a total of 4 × 5-layer T175 flasks), one of which was then treated with HiGom (approx. as LD90) while the other remained an untreated control. Four rounds of selection were performed by treating cells with HiGom for 72 h followed by recovery on regular cell culture media for a further 72 h for each round. Following selection, 2 × 10^7^ cells were pelleted from each group and carried forward for genomic DNA extraction and next-generation sequencing.

### Preparation of samples for next-generation sequencing

Genomic DNA was isolated from cell pellets using the ISOLATE II Genomic DNA Kit (Bioline) according to the manufacturer’s instructions. A two-step PCR was then adapted and used to prepare samples for next-generation sequencing [32]. NEBNext High Fidelity 2x PCR Master Mix (New England Biolabs) was used for all PCRs with primers at final concentrations of 0.4 uM (Cycling conditions listed in Table S2**;** Primer sequences can be found in Table S3). Briefly, 10 ug DNA per sample was used in PCR 1. The product from PCR 1 was then processed using the Bioline ISOLATE II Gel and PCR Kit (Bioline). PCR2 was then performed using 10 ng of PCR1 amplicon as input per reaction. Samples were then loaded onto 2% agarose gels and run at 100 V until clear separation of bands. Bands were then visualized and excised under UV light and were then extracted using the ISOLATE II Gel and PCR kit (Bioline). Samples were then sent to Novogene for next-generation sequencing.

### CRISPR Screen analysis

Cutadapt (v2.6) [33] was used to trim reads using the 5’ adaptor sequence TGTGGAAAGGACGAAACACC. All reads were then processed using MAGeCK (v0.5.9.5) [34] with normalization to control sgRNA. Data was visualized using the MAGeCKFlute (v1.12.0) [33] and enhanced volcano (v1.20.0) R packages [35].

### Ingenuity Pathway Analysis

Ingenuity Pathway Analysis (v1.22.01) [36] was used to analyze results from MAGeCK analysis of CRISPR KO. Core analysis was performed using only genes with positive selection *p*-value < 0.05 and positive selection absolute log_2_ fold change (LFC) greater than 1. Data was exported and visualized using the R packages ggplot2 (v3.4.4) and dplyr (v1.1.4).

### shRNA knock-down

To silence selected target genes (ST3GAL5, B4GAL5, and UGCG) specific shRNA sequences were designed and synthesized using the MISSION® Lentiviral Transduction Particles system (Sigma-Aldrich, St. Louis, MO, USA). More than three distinct shRNAs targeting each gene of interest were selected from the MISSION® shRNA library based on their reported efficacy and specificity, as provided by the manufacturer. Additionally, a non-targeting control shRNA (SHC016-V, scrambled shRNA) for each gene was included to assess non-specific effects. The shRNA sequences were integrated into the pLKO.1-puro lentiviral vector, which contains a puromycin resistance gene for selection. Table S4 specifies the selected shRNAs and their sequence.

MM96L and NFF cells were seeded in complete medium at 50-80% confluency 24 hours prior to transduction in 96-well plates and allowed to adhere overnight. The lentiviral transduction particles were added to the cells at a multiplicity of infection (MOI) of 1 (along with 8 µg/mL polybrene (Sigma-Aldrich). After 24 hours, the medium was replaced with fresh complete medium, and transduced cells were selected using 2 µg/mL puromycin in MM96L cells and 1.5 µg/mL puromycin in NFF cells for 72 hours. Then, cells were expanded for validation by qRT-PCR and MTT.

### RNA extraction and qRT-PCR

Total RNA was isolated from KD cells using a RNeasy kit (Qiagen, #74106), and cDNA synthesis was performed using a SensiFAST™ kit (Bioline, Memphis, TN, USA) according to the following protocol. One microgram of total RNA was reverse transcribed using a total volume of 20 μL. The reaction started at 25 °C for 15 min and 42 °C for 1 h., followed by 85 °C for 5 min. 2 μL of diluted cDNA was used as a template for qPCR amplification by using PowerUp SYBR-green master mix (Thermo Fisher Scientific, Waltham, USA). Quantitative real-time PCR was completed using an Applied Biosystems 7900HT Fast Real-Time PCR System. All reactions were performed in triplicate. The PCR reaction mix was first subjected to 95 °C for 5 min., followed by 45 cycles of amplification. Each cycle consisted of 95 °C for 15 s, annealing temperature of 60 °C for 30 s, and elongation at 72 °C for 1 min. For analysis, gene expression levels were normalized to the mRNA expression levels of the 18S and GAPDH housekeeping genes and expressed relative to the mean of the controls. All primers used in this experiment are listed in Table S5.

### Lipidomics cell culture, homogenization, and sample preparation

MM96L cells and NFF fibroblast were treated with AgGom, HiGom, SpGom and vehicle (PBS) for 12 h and at 70% confluency. Then, cells were resuspended, washed 5 times with cold PBS, and cell pellets were flash-frozen for lipidome analysis. Samples were randomized, homogenized and equalized in homogenization buffer, and lipids were extracted from 10 μL of the homogenized cell culture as previously described [37].

### Lipidomics analysis

Lipids standards list described in Table S6. Lipidic extracts were analyzed via ultra-high-performance liquid chromatography (UHPLC) coupled to electrospray ionization quadrupole time of flight (ESI-Q-TOF) tandem mass spectrometry (MS/MS) (Agilent Technologies). Lipid extracts of 10 μL in the upper phase were injected into 1.8 μm particle 100 × 2.1 mm id Waters Acquity HSS T3 column (Waters, Milford, MA, USA) heated at 55 °C. The flow rate was 400 μL/min with solvent A composed of 10 mM ammonium acetate in acetonitrile-water (40:60, v/v) and solvent B composed of 10 mM ammonium acetate in acetonitrile-isopropanol (10:90, v/v). Data were acquired in both polarities and a pool of all lipid extracts was prepared and used as quality control and instrument bias correction following previously published methods [37, 38].

### Lipidomics data processing and analysis

MassHunter Profinder and Mass Profiler Professional Software (Agilent Technologies) were used to obtain the molecular features of the samples with a minimum of 2 ions and allow one or two charges. Compounds were aligned using a retention time window of 0.1% ± 0.25 min and a mass window of 30 ppm. We selected only those features present in at least 70% of samples from the same group and corrected for individual [39] and signal bias using a LOESS approach [40] developed in-house. Features representing significant differences by linear models were searched at public and in-house libraries and compared to retention time of the authentic standards added and annotated following the scientific guidelines [41, 42] by searching experimental MS data and MS/MS spectra in in silico libraries such as HMDB [43] and LIPID MAPS [44] and by using R-based tools for identification such as LipidMatch [45].

### Lipidomics statistics

The lipid-scaled abundance was analyzed in base 10 logarithmic scale and normalized for composition bias by the trimmed mean of M values (TMM) method [46]. A two-step analysis was applied. First, the subset of lipids showed a significant overall F test for the model with the interaction between both experimental factors and after adjusting for the false discovery rate by Benjamini-Hochberg, they were selected. Then, limited contrasts of differential expressions in this subset were applied. A modeling strategy to fit robust linear models was used. Venn diagrams and model plots present the significantly differentially expressed lipids across comparisons. Measures of dispersion included the coefficients of variation and the quartile coefficients of dispersion. All the analysis was performed in R, applying a significance level of 0.05.

### Animal studies

Female seven-week-old NOD.Cg-Prkdc<scid> Il2rg<tm1Wjl>SzJ in-house breeding but originally purchased by Jackson lab, were housed in strict animal husbandry facilities free of pathogens and micro-organisms. The facilities had a controlled temperature environment and normal 12-hour day-night light cycles. In brief, animals were randomly selected for peptide or vehicle treatment and placed in two cages of five animals each. Each cage (containing five animals) had bedding to provide shelter and privacy as well as unlimited access to food and water. No blinding was performed during the animal experiments. Animals were inspected daily by qualified personnel and weekly or as needed by the institute’s veterinary doctor. At the end of the experiments, mice were sacrificed by CO_2_ followed by cervical dislocation.

### Xenograft studies

We used five mice per group and injected subcutaneously 2.5 million cells of MM96L control cells (groups 1 and 2) or MM96L ST3GAL5 KD cells (groups 3 and 4) in both mouse flanks. Maximal tolerable dose assays have previously been determined [7] in which the HiGom dose was safely incremented up to 15 mg/kg. For this study, vehicle or HiGom at 10 mg/kg were administered i.p. every 2 days for 14 days (7 injections). Tumor progression was evaluated as described before [7, 47].

### Data analysis

The declared group size is the number of independent values, and statistical analysis was done using these independent values. The effects of HiGom were not predictable in vivo; hence, a sample size estimation was not performed, and experiments were evaluated to comply with the 3 R principles (replace, reduce, and refine). In addition, statistical analysis was undertaken for studies where each group size was at least *n* = 3. All data were performed in triplicate and are expressed as the mean ± standard error of the mean deviation (SEM). Statistical analyses were conducted using either a Student’s *t*-test if only two variables were tested (e.g., control and peptide-treated samples) or one-way ANOVA followed by a Dunnet’s post hoc test for multiple comparisons. Statistical significance was considered at *P < 0.05. Of note, in multigroup studies with parametric variables, post hoc tests were conducted only if F in ANOVA (or equivalent) achieved the necessary level of statistical significance (*P* < 0.05) and there was no significant variance in homogeneity. All sample populations were first checked for normality. When populations within groups were not normally distributed, a non-parametric Mann-Whitney test was used for single comparisons or the Kruskal-Wallis test for multiple comparisons. IC_50_ values were calculated using a non-linear regression with the model log(inhibitor) vs. normalized response and least squares fit. *P < 0.05 was considered statistically significant. To control unwanted sources of variation between individual experiments, data was normalized to the mean of control as Prism 9.

## Supplementary information

Supplementary file

## Data Availability

All data supporting this work are available within this article and the supplementary section or from the corresponding author upon request.
