# Correction: The cytotoxicity of gomesin peptides is mediated by the glycosphingolipid pathway and lipid-cholesterol interactions

**DOI:** 10.1038/s41420-026-03009-x

**Published:** 2026-04-02

**Authors:** Isabel Fernandez-Carrasco, Javier Moral-Sanz, Sergey Kurdyukov, Èlia Obis, Lissy Maike Hartmann, Silvia Carina Magalhães Novais, Matthew A. Waller, Naomi McKinnon, Felicity Chung, Francisco Javier Salazar Castejón, Daniel P. Rainho, Zoltan Dekan, Thomas Kremsmayr, Bernhard Jandl, Kristina Eleršič Filipič, Reinald Pamplona, Mariona Jové, Manuel A. Fernandez-Rojo, Gregor Anderluh, Markus Muttenthaler, Paul F. A. Alewood, Jan Procházka, G. Gregory Neely, Evelyne Deplazes, Maria P. Ikonomopoulou

**Affiliations:** 1 Madrid Institute for Advanced Studies in Nutrition (IMDEA Nutrition), Madrid, Spain; 2 Dr. John and Anne Chong Lab for Functional Genomics, Charles Perkins Centre and School of Life & Environmental Sciences, The University of Sydney, Sydney, NSW Australia; 3 Department of Experimental Medicine, Lleida Biomedical Research Institute (IRB Lleida), University of Lleida (UdL), Lleida, Spain; 4 School of Chemistry and Molecular Biosciences, The University of Queensland, St. Lucia, QLD Australia; 5 Czech Centre for Phenogenomics, Institute of Molecular Genetics of the Czech Academy of Sciences, Vestec, Czechia; 6 Institute for Molecular Bioscience, The University of Queensland, Brisbane, QLD Australia; 7 Institute of Biological Chemistry, University of Vienna, Vienna, Austria; 8 Department of Molecular Biology and Nanobiotechnology, National Institute of Chemistry, Ljubljana, Slovenia; 9 Frazer Institute, Translational Research Institute, The University of Queensland, Woolloongabba, QLD Australia; 10 School of Life Sciences, University of Technology Sydney, Ultimo, NSW Australia

**Keywords:** Melanoma, Biologics

Correction to: *Cell Death Discovery* 10.1038/s41420-025-02817-x, published online 21 November 2025

In this article the author name should appear as “Obis E” instead of “Monné ÈO”. Author name: Elia, Author surname: Obis.

The original article has been corrected.

