# Supplementary material for: The cytotoxicity of gomesin peptides is mediated by the glycosphingolipid pathway and lipid-cholesterol interactions
Source: Cell Death Discov. 2025 Nov 21;11:538. doi: 10.1038/s41420-025-02817-x (PMC12638847; doi:10.1038/s41420-025-02817-x)
Supplement: Supplementary file 1 — Supplementary file [file 41420_2025_2817_MOESM1_ESM.pdf]

Supplementary file

The cytotoxicity of gomesin peptides is mediated by the glycosphingolipid pathway and lipid-cholesterol interactions

Isabel Fernandez-Carrasco[1], Javier Moral-Sanz[1], Sergey Kurdyukov[2], Èlia Obis Monne[3], Lissy Maike Hartmann[4], Silvia Carina Magalhães Novais[5], Matthew A. Waller[2], Naomi McKinnon[2], Felicity Chung[2], Francisco Javier Salazar Castejón[1], Daniel P. Rainho[1], Zoltan Dekan[6], Thomas Kremsmayr[7], Bernhard Jandl[6,7], Kristina Eleršič Filipič[8], Reinald Pamplona[3], Mariona Jové[3], Manuel A. Fernandez-Rojo[1,9], Gregor Anderluh[8], Markus Muttenthaler[6,7], Paul F. A. Alewood[6], Jan Procházka[5], G. Gregory Neely[2], Evelyne Deplazes[4,10], Maria P. Ikonomopoulou[1,6*].

[1] Madrid Institute for Advanced Studies in Food (IMDEA Nutrition), Madrid, E28049, Spain
[2]Dr. John and Anne Chong Lab for Functional Genomics, Charles Perkins Centre and School of Life & Environmental Sciences, The University of Sydney, Sydney, NSW 2006, Australia.
[3]Department of Experimental Medicine, Lleida Biomedical Research Institute (IRB Lleida), University of Lleida (UdL), 25198 Lleida, Spain.
[4]School of Chemistry and Molecular Biosciences, The University of Queensland, St. Lucia, QLD 4072, Australia
[5]Czech Centre for Phenogenomics, 25250 Vestec, Czech Rebublic
[6]Institute for Molecular Bioscience, The University of Queensland, 4072 Brisbane, Australia
[7]Institute of Biological Chemistry, University of Vienna, 1090 Vienna, Austria
[8]Department of Molecular Biology and Nanobiotechnology, National Institute of Chemistry, SI-1000 Ljubljana, Slovenia.
[9]Frazer Institute, Translational Research Institute, The University of Queensland, Woolloongabba, QLD 4102, Australia.
[10]School of Life Sciences, University of Technology Sydney, Ultimo, NSW, 2007, Australia

*Correspondence: maria.ikonomopoulou@nutricion.imdea.org

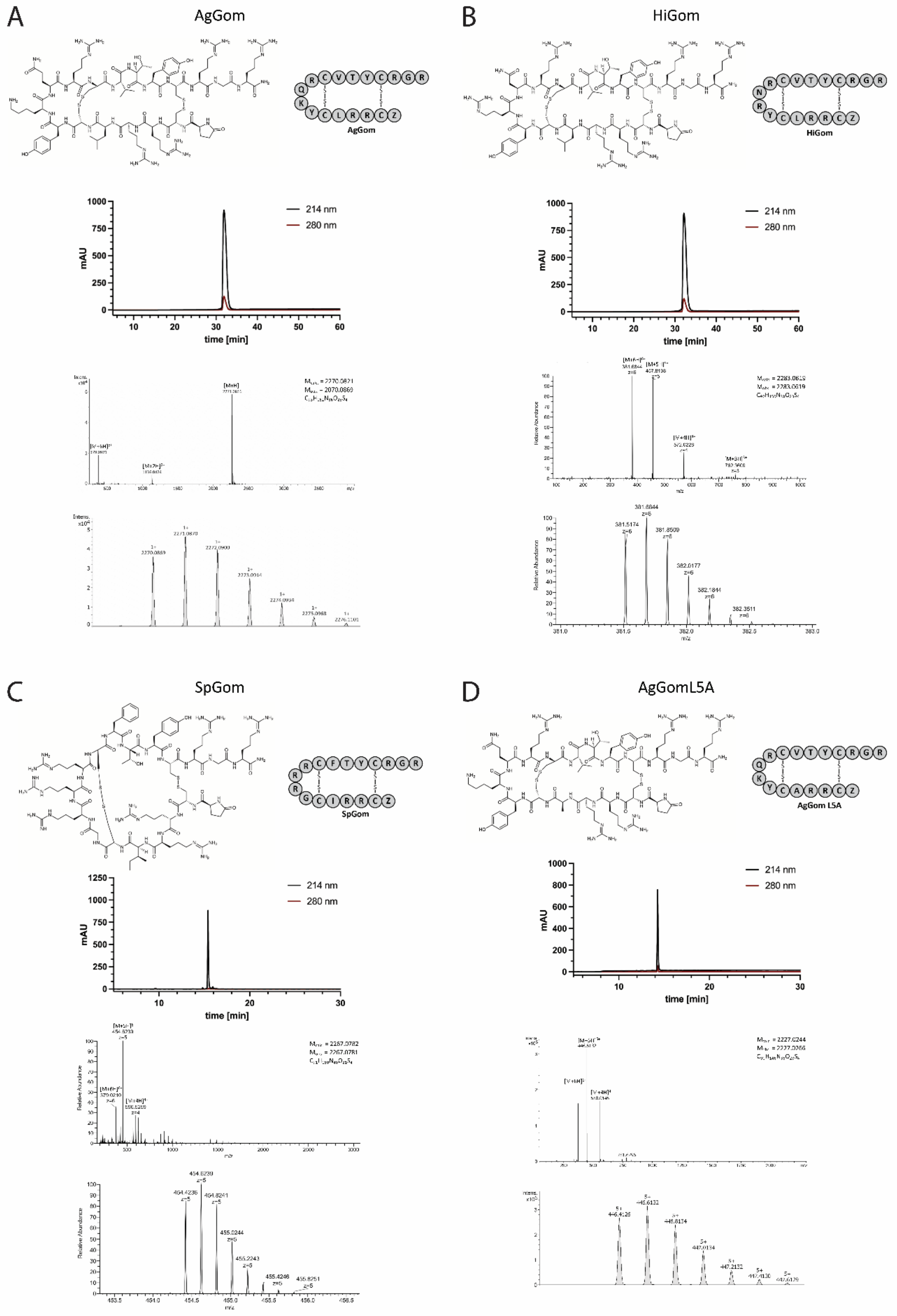

Figure S1. Gomesin compounds characterisation sheet (**A-D**), including the chemical structure, peptide sequence, analytical RP-HPLC chromatogram and HRMS data.

Table S1. List of calculated and observed monoisotopic masses for all gomesin analogs.

Name	Sequence	Mass calc	Mass obs
AgGom	$ZC_1RRLC_2YKQRC_2VTYC_1RGR$*	2270.0821	2070.0869
HiGom	$ZC_1RRLC_2YRNRC_2VTYC_1RGR$*	2283.0619	2283.0619
SpGom	$ZC_1RRIC_2GRRRC_2FTYC_1RGR$*	2267.0782	2267.0781
AgGomL5A	$ZC_1RRAC_2YKQRC_2VTYC_1RGR$*	2227.0244	2227.0266

* C-terminal amide; disulfide connectivity: C_1-C_1; C_2-C_2

Note: Z is a pyroglutamic acid residue

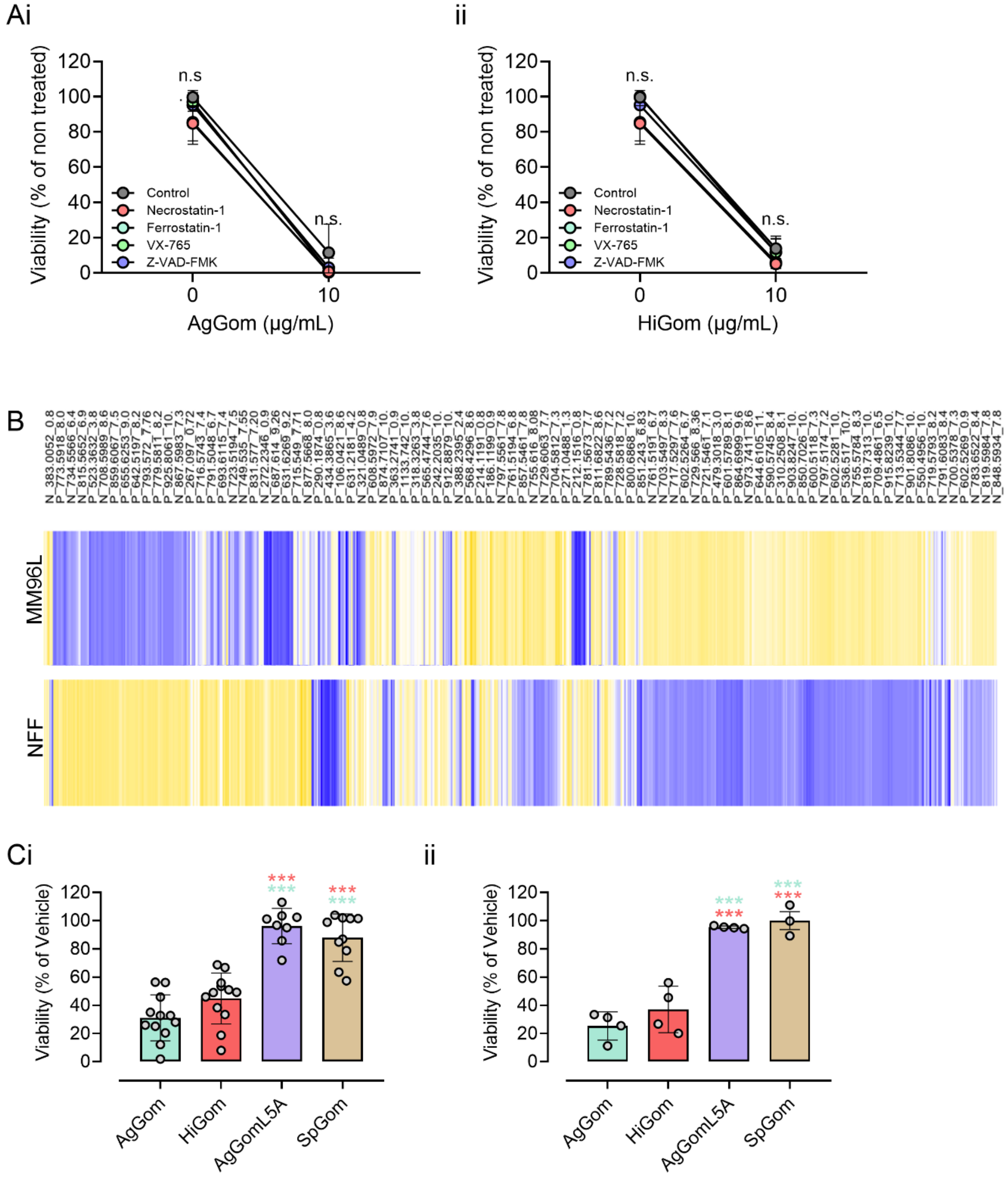

Figure S2. (A) Viability of MM96L cells analysed with crystal violet for the effects of **(i)** AgGom and **(ii)** HiGom under control condition (grey) or in the presence of a necroptosis inhibitor (pink, 50µM Necrostatin-1), a ferroptosis inhibitor (blue, 30µM Ferrostatin-1), a caspase-1/4 inhibitor (green, 20 µM VX-765) or a pan-caspase inhibitor (purple, 30µM z-VAD FMK) (n=3). **(B)** Lipidomic analysis with a total of 1703 lipids found in MM96L and NFF cells. **(C)** Viability of HiGom (red), AgGom (blue), AgGomL5A (purple) and SpGom (brown) peptides at **(i)** 10 µg/mL in MM96L cells and **(ii)** 30 µg/mL in NFF cells. Data are shown as mean ± SEM. Statistical significance was assessed by one-way ANOVA test with Šídák's correction in panels A (n.s.= no significance) and C, respectively. ***p < 0.001 vs AgGom treatment (blue) and vs HiGom treatment (red).

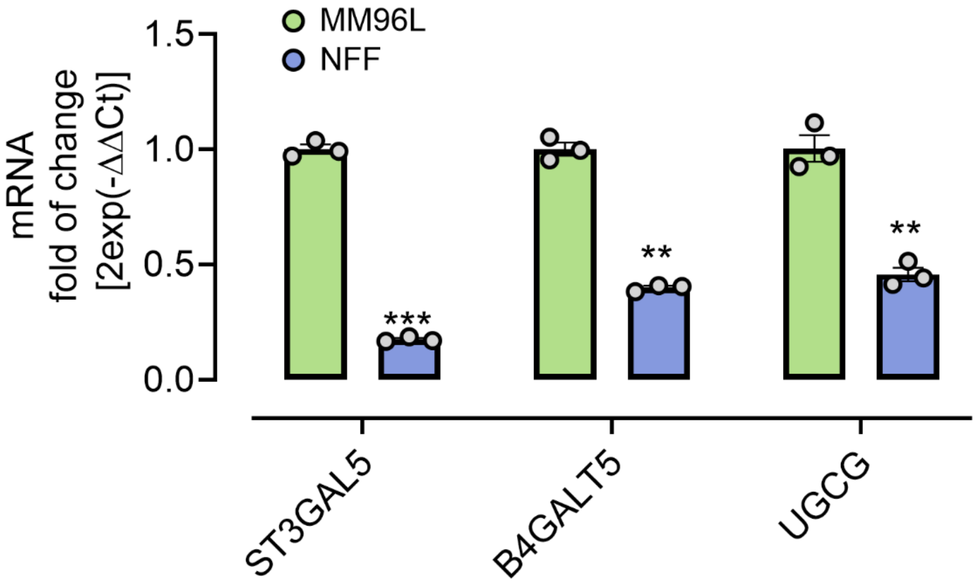

Figure S3. Gene expression of ST3GAL5, B4GALT5, SAP30BP, UGP2, and UGCG was measured by quantitative PCR and normalized using the 2^(-ΔΔCt) method in MM96L (green) and NFF (purple) cells. Each bar represents the mean ± SEM (n=3), with individual data points shown. Statistical comparisons were performed using an unpaired t-test (**p < 0.01 and ***p < 0.001 vs MM96L cells).

Table S2. PCR Cycling Conditions (CRISPR/Cas9)

Number of cycles	Step	Temperature (ºC)	Time
**PCR 1**			
1	Initial denaturation	98	1 min
20	Denaturation	98	20 s
	Annealing	66	30 s

	Extension	72	30 s
1	Final extension	72	2 min
**PCR 2**			
1	Initial denaturation	98	1 min
3	Denaturation	98	20 s
	Annealing	66	30 s
	Extension	72	30 s
15	Denaturation	98	20 s
	Annealing & Extension	72	30 s
1	Final extension	72	5 min
N/A	Hold	10	Indefinitely

Table S3. Primers used for NGS

Primer	Sequence
	PCR 1
Fwd	GGACAGCAGAGATCCAGTTTGGT
Rev	GAGCCAATTCCCACTCCTTTCAA
	PCR2
i5_Stagger_0	AATGATACGGCGACCACCGAGATCTACACTCTTTCCCTACACGACGCTCTTCCGATCTCTTGTGGAAAGGACGAAACACC
i5_Stagger_1	AATGATACGGCGACCACCGAGATCTACACTCTTTCCCTACACGACGCTCTTCCGATCTGTCTTGTGGAAAGGACGAAACACC
i5_Stagger_2	AATGATACGGCGACCACCGAGATCTACACTCTTTCCCTACACGACGCTCTTCCGATCTAGTCTTGTGGAAAGGACGAAACACC
i5_Stagger_3	AATGATACGGCGACCACCGAGATCTACACTCTTTCCCTACACGACGCTCTTCCGATCTGCCTCTTGTGGAAAGGACGAAACACC
i5_Stagger_4	AATGATACGGCGACCACCGAGATCTACACTCTTTCCCTACACGACGCTCTTCCGATCTACAATCTTGTGGAAAGGACGAAACACC
i5_Stagger_5	AATGATACGGCGACCACCGAGATCTACACTCTTTCCCTACACGACGCTCTTCCGATCTTAGAGTCTTGTGGAAAGGACGAAACACC
i5_Stagger_6	AATGATACGGCGACCACCGAGATCTACACTCTTTCCCTACACGACGCTCTTCCGATCTCAGCAATCTTGTGGAAAGGACGAAACACC
i5_Stagger_7	AATGATACGGCGACCACCGAGATCTACACTCTTTCCCTACACGACGCTCTTCCGATCTTGAGACATCTTGTGGAAAGGACGAAACACC
i7_index_1	CAAGCAGAAGACGGCATACGAGATGCCAATGTGACTGGAGTTCAGACGTGTGCTCTTCCGATCTACCGACTCGGTGCCACTTTTTCAAG
i7_index_2	CAAGCAGAAGACGGCATACGAGATCTTGTAGTGACTGGAGTTCAGACGTGTGCTCTTCCGATCTACCGACTCGGTGCCACTTTTTCAAG
i7_index_3	CAAGCAGAAGACGGCATACGAGATAGTTCCGTGACTGGAGTTCAGACGTGTGCTCTTCCGATCTACCGACTCGGTGCCACTTTTTCAAG
i7_index_4	CAAGCAGAAGACGGCATACGAGATATGTCAGTGACTGGAGTTCAGACGTGTGCTCTTCCGATCTACCGACTCGGTGCCACTTTTTCAAG

Table S4. showing the selected shRNAs and their sequence.

Gene	ID shRNA	Sequence
ST3GAL5 (RefSeq NM_003896)	TRCN0000036137	GTGGAGGCATTGATCGTGAAT
B4GALT5 (RefSeq NM_004776)	TRCN0000035363	CCTCAACAACCTGAACTACTT
UGCG (RefSeq NM_003358)	TRCN0000036128	GCAGAGGAAATCCTAGATGTA

Table S5. Forward and Reverse sequences of six key genes used.

Gene	Forward	Reverse
hST3GAL5	ACCCTGCCATTCTGGGTACG	ACACCGATTGTGGGGACGTT
hB4GALT5	CTCGTCCTCGCTGCTGTACT	ACTCCGAAGCACCTGCTCAT
hCYBRD1	CGGCTTCCTGTCGGTGATCTT	CCACGGCAGTCTGTAGACGA
hSAP30BP	TGATGCCTATGGGGAGGATGA	AAAAGGAGGCCACGAGTTCCT
hUGCG	GGTTCGTCCTCTTCTTGGTGC	GGCTGTTTGTCAGTTGCCTTCT
hUGP2	CCTGCCCTGTAGCGTGACT	GCCGAATGACTTCTTGGAACTG

Table S6. Lipids

Class representative internal standards. All lipid standards are acquired from Avanti Polar Lipids except Stearic acid, which was purchased from Merck, and stock solutions were prepared by dissolving standards at a concentration of 1 mg/mL and working solutions were diluted to 2.5 µg/ mL in MTBE.

**Compound**	**Reference**
1,3(d5)-dihexadecanoyl-2-octadecanoyl-glycerol	860902
1-palmitoyl-d31-2-oleoyl-sn-glycero-3-phosphate	860453

1-hexadecanoyl-2-(9Z-octadecenoyl)-sn-glycero-3-phospho-(1′-rac-glycerol-1′,1′,2′,3′,3′-d5)	860385
1-palmitoyl-d31-2-oleoyl-sn-glycero-3-phosphoethanolamine	860374
1-palmitoyl-d31-2-oleoyl-sn-glycero-3-phosphoinositol	860042
1-palmitoyl-d31-2-oleoyl-sn-glycero-3-[phospho-L-serine]	860403
1-hexacosanoyl(12,12,13,13-D4)-sn-glycero-3-phosphocholine	860389
25,26,26,26,27,27,27-heptadeuteriocholest-5-en-3β-ol (9Z-octadecenoate)	700185
cholest-5-en-3β-ol(d7)	LM4100
D-erythro-sphingosine-d7-1-phosphate	860659
D-erythro-sphingosine-d7	860657
N-palmitoyl-d31-D-erythro-sphingosine	868516
N-palmitoyl-d31-D-erythro-sphingosylphosphorylcholine	868584
Stearic acid-D35	448249
1,3-Dioctadecanoyl-2-hydroxy-sn-glycerol-d5	800855
